# Renal cell carcinoma induces interleukin 10 and prostaglandin E_2_ production by monocytes

**DOI:** 10.1038/sj.bjc.6690021

**Published:** 1999-09-24

**Authors:** C Ménétrier-Caux, C Bain, M C Favrot, A Duc, J Y Blay

**Affiliations:** Unité Cytokine et Cancer, Centre Léon Bérard, 28 rue Laënnec 69373 Lyon Cedex 08, France; Department of Tumour Biology, Centre Léon Bérard, 69373 Lyon Cedex 08, France

**Keywords:** interleukin 10, prostaglandin E_2_, tumour necrosis factor α, renal cell carcinoma, immunosuppression

## Abstract

Immunotherapy with interleukin 2 (IL-2) is not an effective anti-cancer treatment in the majority of patients with renal cell carcinoma (RCC), suggesting that the activation of cytotoxic T cells or NK cells may be impaired in vivo in these patients. The production of immunosuppressive factors by RCC was investigated. Using immunohistochemistry, IL-10 was detectable in 10 of 21 tumour samples tested. IL-10 was undetectable in the supernatant of cell lines derived from these RCCs. However, these cell lines or their conditioned medium (RCC CM), but not normal renal epithelial cells adjacent to the RCC or breastcarcinoma cell lines, were found to induce IL-10, as well as prostaglandin E_2_ (PGE_2_) and tumour necrosis factor (TNF)α production by autologous or allogeneic peripheral blood mononuclear cells (PBMCs) and monocytes. IL-10 production induced by RCC CM was found to be dependent on TNF-α and PGE_2_ since an anti-TNF-α antibody (Ab) inhibited 40–70% of IL-10 production by monocytes, and the combination of anti-TNF-α Ab and indomethacin, an inhibitor of PGE_2_ production, inhibited 80–94% of RCC CM-induced IL-10 production by monocytes. The RCC CM of the five cell lines tested were found to induce a down-regulation of the expression of HLA-DR and CD86, as well as a strong inhibition of mannose receptor-dependent endocytosis by monocytes. The blockade of HLA-DR and CD86 expression was partially abrogated by indomethacin and anti-IL-10 Ab respectively, and completely abrogated by an anti-TNF-α Ab. The inhibition of mannose receptor-dependent endocytosis was partially abrogated by an anti-IL-10 Ab and completely abrogated by an anti-TNF-α Ab. These esults indicate that RCCs induce IL-10, PGE_2_ and TNF-α production by monocytes, which down-regulate the expression of cell-surface molecules involved in antigen presentation as well as their endocytic capacity. © 1999 Cancer Research Campaign


*British Journal of Cancer* (1999) **79**(1), 119–130
© 1999 Cancer Research Campaign

### Renal cell carcinoma induces interleukin 10 and prostaglandin $E_2$ production by monocytes

**C Ménétrier-Caux[1], C Bain[2], MC Favrot[2], A Duc[2] and JY Blay[1]**

[1]Unité Cytokine et Cancer, Centre Léon Bérard, 28 rue Laënnec, 69373 Lyon Cedex 08, France; [2]Department of Tumour Biology, Centre Léon Bérard, 69373 Lyon Cedex 08, France

**Summary** Immunotherapy with interleukin 2 (IL-2) is not an effective anti-cancer treatment in the majority of patients with renal cell carcinoma (RCC), suggesting that the activation of cytotoxic T cells or NK cells may be impaired in vivo in these patients. The production of immunosuppressive factors by RCC was investigated. Using immunohistochemistry, IL-10 was detectable in 10 of 21 tumour samples tested. IL-10 was undetectable in the supernatant of cell lines derived from these RCCs. However, these cell lines or their conditioned medium (RCC CM), but not normal renal epithelial cells adjacent to the RCC or breast carcinoma cell lines, were found to induce IL-10, as well as prostaglandin $E_2$ ($PGE_2$) and tumour necrosis factor (TNF)α production by autologous or allogeneic peripheral blood mononuclear cells (PBMCs) and monocytes. IL-10 production induced by RCC CM was found to be dependent on TNF-α and $PGE_2$ since an anti-TNF-α antibody (Ab) inhibited 40–70% of IL-10 production by monocytes, and the combination of anti-TNF-α Ab and indomethacin, an inhibitor of $PGE_2$ production, inhibited 80–94% of RCC CM-induced IL-10 production by monocytes. The RCC CM of the five cell lines tested were found to induce a down-regulation of the expression of HLA-DR and CD86, as well as a strong inhibition of mannose receptor-dependent endocytosis by monocytes. The blockade of HLA-DR and CD86 expression was partially abrogated by indomethacin and anti-IL-10 Ab respectively, and completely abrogated by an anti-TNF-α Ab. The inhibition of mannose receptor-dependent endocytosis was partially abrogated by an anti-IL-10 Ab and completely abrogated by an anti-TNF-α Ab. These results indicate that RCCs induce IL-10, $PGE_2$ and TNF-α production by monocytes, which down-regulate the expression of cell-surface molecules involved in antigen presentation as well as their endocytic capacity.

**Keywords:** interleukin 10; prostaglandin $E_2$; tumour necrosis factor α; renal cell carcinoma; immunosuppression

Although immunotherapy with IL-2 and/or interferon α (IFN-α) yields objective response in 15% of patients with metastatic renal cell carcinoma, a majority of these patients will still experience progressive disease despite immunotherapy (Rosenberg et al, 1989; Négrier et al, 1996). This may result from the lack of tumour-specific antigens in most of these tumours, a defective tumour-specific antigen presentation and/or the production of immunosuppressive factors by RCC.

IL-10 is a potent immunosuppressive cytokine produced by B lymphocytes, monocytes/macrophages as well as Th0 and Th2 T-cell subsets (Howard and O’Garra, 1992). IL-10 inhibits the proliferation and the production of IL-2 and IFN-γ by human peripheral blood T lymphocytes and T-cell clones (de Waal Malefyt et al, 1993; Taga et al, 1993) and suppresses the secretion of IL-1, IL-6, IL-8 and TNF-α (Fiorentino et al, 1991). In addition, IL-10 induces the generation of $CD4^+$ T cells that are capable of blocking the proliferation of Ag-specific T-cell clones (Groux et al, 1997). The production of IL-10 mRNA or protein has been reported in RCC, as well as in other tumours, in particular ovarian tumours and non-Hodgkin’s lymphoma (NHL), suggesting a possible local immunosuppressive effect of this cytokine in vivo (Benjamin et al, 1992; Emilie et al, 1992; Pisa et al, 1992; Blay et al, 1993; Filgueira et al, 1993; Gastl et al, 1993; Mauerer et al, 1995; Wang et al, 1995; Nakagomi et al, 1995; Voorzanger et al, 1996).

*Received 3 November 1997*
*Revised 28 January 1998*
*Accepted 3 March 1998*

*Correspondence to:* JY Blay

The objectives of this study were to investigate the possible production of immunosuppressive mediators by RCC tumours, in particular IL-10. The results presented here show that IL-10 protein is detectable in vivo in RCC tumour samples and that RCC cell lines induce the production of TNF-α, IL-10 and $PGE_2$ by autologous and allogeneic monocytes in vitro. TNF-α, IL-10 and $PGE_2$ are responsible for the down-regulation of HLA-DR and CD86 expression at the cell surface, as well as for the decrease in the endocytic capacities of monocytes induced by RCC CM in vitro.

### MATERIALS AND METHODS

### Cell lines and tumour samples

Clear cell renal carcinoma cells were obtained after therapeutic surgery of the primary tumour in 21 patients. Each tumour sample was divided into four fragments, representative of the tumour, as evaluated by examination of a frozen section, for (1) histological analysis, (2) molecular analysis, (3) storage in isopentane at –180°C for immunological analysis and (4) mechanical and enzymatic disaggregation to obtain tumour cells for culture. Four RCC cell lines (CAN, CHA, GUI, VER) previously characterized (Bain et al, 1996) and two primary cell cultures (LEC, DUF) were obtained from these 21 tumours. Primary culture of normal renal epithelial cells (CAN nor) was obtained after enzymatic disaggregation of normal tissue surrounding the tumour. In addition, RCC cell lines (Caki-1, Caki-2, ACHN, A-704) and breast carcinoma cell lines used as control (SKBR-3, T47-D, MCF-7) were obtained from the American Type Cell Collection (ATCC) (Rockville, MD, USA). All cell lines were cultured in complete RPMI-1640

medium supplemented with 2 mM glutamine, 200 U ml$^{-1}$ penicillin, 200 μg ml$^{-1}$ streptomycin (Gibco Laboratories, Grand Island, NY, USA) and 10% fetal calf serum (Biowittaker, Verviers, Belgium). All these cell lines are regularly tested for mycoplasma infection using a specific enzyme-linked immunosorbent assay (ELISA) (Boehringer Mannheim, Meylan, France) and consistently found to be negative.

### PBMC, monocytes and lymphocytes preparation

Total peripheral blood mononuclear cells (PBMCs) were isolated from heparinized blood obtained from healthy volunteers and from four patients with RCC in whom tumour cell lines (CAN, VER) or tumour cell cultures (LEC, DUF) were obtained, using Ficoll–Hypaque density-gradient centrifugation (Eurobio, Les Ulis, France). Monocytes and lymphocytes were further purified on a multistep Percoll gradient as previously described (Sallusto et al, 1994). Around 85–90% of the cells in the monocyte-enriched fraction were positive for CD14 expression using flow cytometry, compared with less than 3% of the cells in the lymphocyte-enriched population.

### Conditioned medium (CM) of renal cell carcinoma and other cell lines

Carcinoma cell lines were plated in 100-mm-diameter Petri dishes at a density of $5 \times 10^5$ cells ml$^{-1}$ in complete medium. After 2 days of culture, supernatants were harvested, filtered through 0.22-μm mesh, aliquoted and stored at –20°C for further investigations. Each batch was tested for mycoplasma contamination and found to be negative.

### Culture conditions

PBMCs, monocytes and lymphocytes were cultured in RPMI with 10% FCS in 24-well flat-bottomed plates (Falcon Labware, Oxnard, CA, USA) for the quantification of IL-10 production. PBMCs ($10^6$), $4 \times 10^5$ monocytes and $8 \times 10^5$ lymphocytes were plated in a final volume of 1 ml with $5 \times 10^4$ tumoral cells. In transwell (Costar, Brumath, France) culture conditions, monocytes were seeded in the upper compartment and tumoral cells in the lower compartment of 24-well plates. Cultures with conditioned medium (CM) were performed by addition of 5–80% or 40% RCC CM according as specified. The positive control for IL-10 production was obtained by addition of lipopolysaccharide (LPS), (1 μg ml$^{-1}$) to PBMCs, monocytes or lymphocytes culture. Cells cultured in RPMI with 10% FCS alone were used as negative control.

Supernatants were collected after 48 h as this time has been reported to be the peak of IL-10 production by monocytes (de Waal Malefyt et al, 1991).

### Reagents

Purified human cytokines were used at the indicated concentrations: rIL-1β (100 units per ml) was purchased from Innogenetics (Zwijnaarde, Belgium); rTNF-α (100 ng ml$^{-1}$), IL-6 (100 ng ml$^{-1}$), basic Fibroblast growth factor (FGFb) (0.1–100 ng ml$^{-1}$) and TGF-β (1–100 ng ml$^{-1}$) from Genzyme Corporation (Boston, MA, USA) and granulocyte–macrophage colony-stimulating factor (GM-CSF) (1–100 ng ml$^{-1}$) and-IL-10 from Schering Plough Corporation (Dardilly, France). Neutralizing anti-IL-10 rabbit polyclonal antibody was kindly provided by N Burdin (Schering Plough Corporation, Dardilly, France). The anti-IL-10 monoclonal antibody (Ab85) used in immunohistochemistry was kindly provided by I Joab and used at a 1:200 final concentration (Emilie et al, 1992; Voorzanger et al, 1996). Neutralizing anti-TNF-α polyclonal antibody (IP-300) was purchased from Genzyme Corporation and used at 10 μg ml$^{-1}$ LPS from *Escherichia coli* serotype 0111:B4 was used at 1 μg ml$^{-1}$ (Sigma, St Louis, MO, USA). Anti-HLA-DR, anti-CD14, anti-CD80, -CD45, -CD3 and -CD20 were purchased from Becton Dickinson, Pont de Claix, France, anti-CD54 from Immunotech, Luminy, France and CD86, anti-IL-10 (9D7) and rat isotype control from Pharmingen, San Diego, CA, USA. G250, which stains specifically RCC tumour cells (Oosterwijk et al, 1986), was kindly provided by Dr Fleuren (Leiden, the Netherlands). Indomethacin (Sigma) was used at the optimal concentration of 10 μM (Kambayashi et al, 1995). The isotype control antibody for anti-IL-10 antibody (Ab85) was a mouse IgG (R & D, Abingdon, UK; 10 μg ml$^{-1}$). The isotype control antibody for anti-TNF-α and anti-IL-10 antibodies used in culture neutralization experiments was a rabbit polyclonal antibody (R & D, 10 μg ml$^{-1}$).

**Table 1** Cytokine pattern in supernatant of culture with RCC CM

| | TNF-α (ng ml$^{-1}$) | PGE-$_2$ (ng ml$^{-1}$) | IL-10 (ng ml$^{-1}$) |
|---|---|---|---|
| Conditioned medium (CM) | | | |
| CAN | 0.011 | < 0.016 | < 0.04 |
| CHA | 0.040 | < 0.016 | < 0.04 |
| GUI | 0.030 | < 0.016 | < 0.04 |
| VER | < 0.01 | < 0.016 | < 0.04 |
| ACHN | < 0.01 | < 0.016 | < 0.04 |
| A-704 | < 0.01 | < 0.016 | < 0.04 |
| Caki-1 | 0.080 | < 0.016 | < 0.04 |
| T47-D | < 0.01 | < 0.016 | < 0.04 |
| CM with monocytes | | | |
| CAN | 0.53 | 0.42 | 0.77 |
| CHA | 1.3 | 1.05 | 0.63 |
| GUI | 1.18 | 0.6 | 0.37 |
| VER | 0.95 | 0.95 | 0.35 |
| A-704 | 0.54 | 1.37 | 1.21 |
| ACHN | 0.4 | 3.25 | 0.75 |
| Caki-1 | 0.08 | 4.7 | 1.03 |
| T-47D | 0.05 | 0.016 | < 0.04 |
| None | 0.054 | 0.02 | < 0.04 |
| LPS | 2.6 | 3.5 | 1.13 |

### Immunohistochemistry

Immunochemical stainings were performed using an indirect three-step immunoenzymatic procedure with alkaline phosphatase (AP) (Combaret et al, 1989). Briefly, air-dried cryostat sections were fixed for 10 min in 4% paraformaldehyde at 4°C and incubated for 30 min with the primary antibody. After two washes in Tris-buffered, saline (TBS) containing 0.2% bovine serum albumin (BSA), slides were incubated for 30 min with AP-conjugated rabbit anti-mouse immunoglobulins (Ig) (Dako, Trappes, France), washed and incubated for 30 min with an AP-conjugated swine anti-rabbit Ig (Dako). Revelation was performed using the AP substrate (naphthol AS MX phosphate, dimethyl formamide, levamisole and fast red), before counterstaining with haematoxylin.

 © Cancer Research Campaign 1999

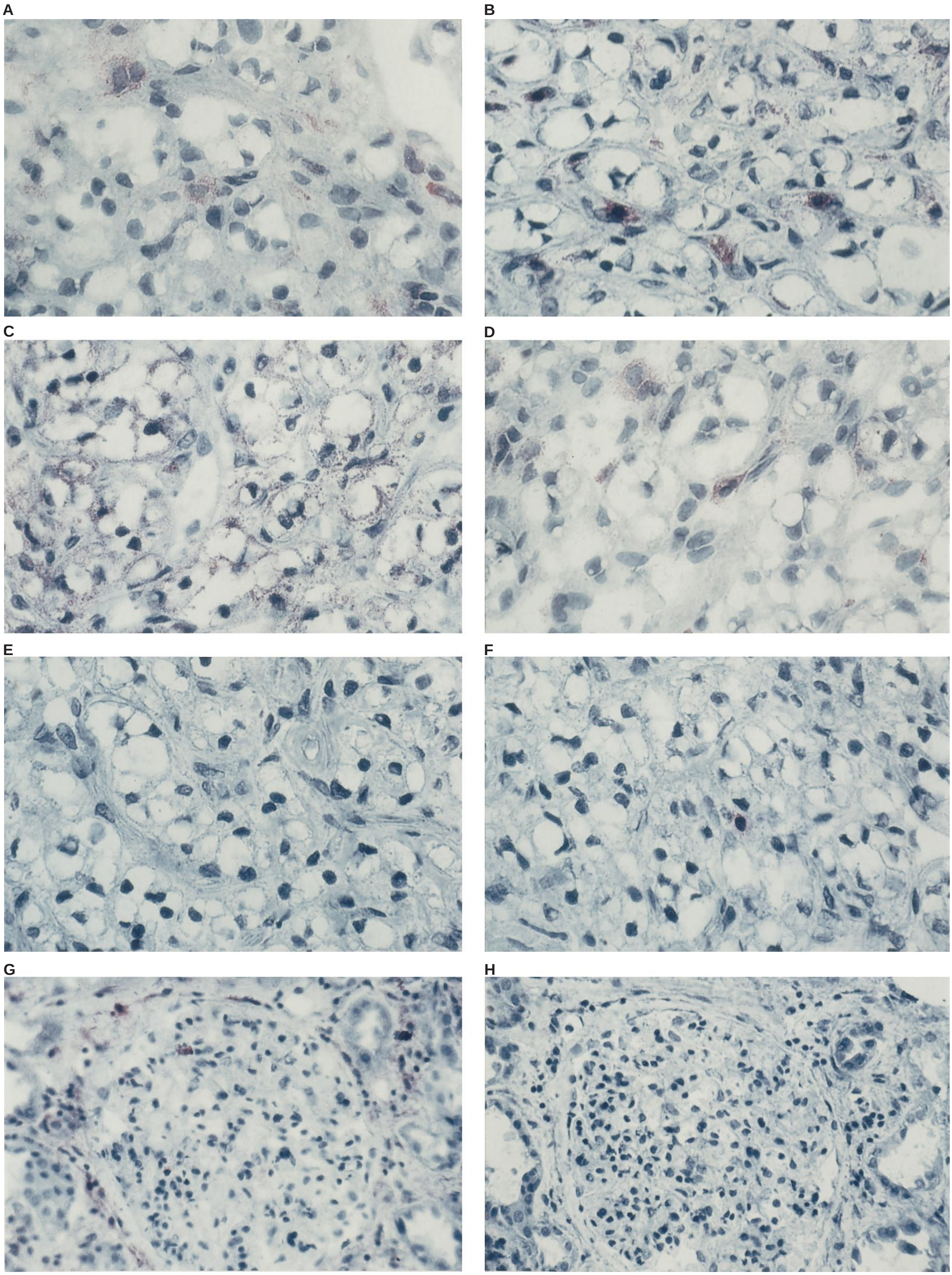

**Figure 1** RCC cryostat sections immunostaining (**A**–**F**). The antibodies used were Ab85 (anti-IL-10) (**A**), 2D1 (CD45) (**B**), G250 (specific for RCC tumoral cells) (**C**), My-4 (CD14) (**D**), B1 (CD20) (**E**) and T3 (CD3) (**F**). The isotype-matched control showed the absence of background staining (data not shown). Negative staining for IL-10 (**H**) in $CD14^+$ (**G**) cells of normal renal parenchyma

*© Cancer Research Campaign 1999*

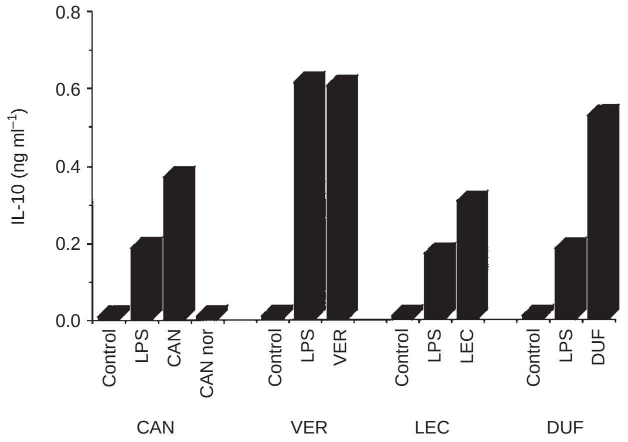

**Figure 2** Mean IL-10 production in co-culture of autologous PBMCs ($10^6$ cells ml$^{-1}$) with four different RCC cell lines (CAN tum, VER, LEC, DUF) or with normal renal epithelial cells (CAN nor) ($5 \times 10^4$ cells per well). Positive control was performed using LPS for each patient. This experiment is representative of three performed with different batch of PBMCs. IL-10 secretion by PBMCs treated with LPS or co-cultured with RCC CM is superior to that of PBMCs cultured with normal renal epithelial cells (Student's *t*-test $P = 0.001$)

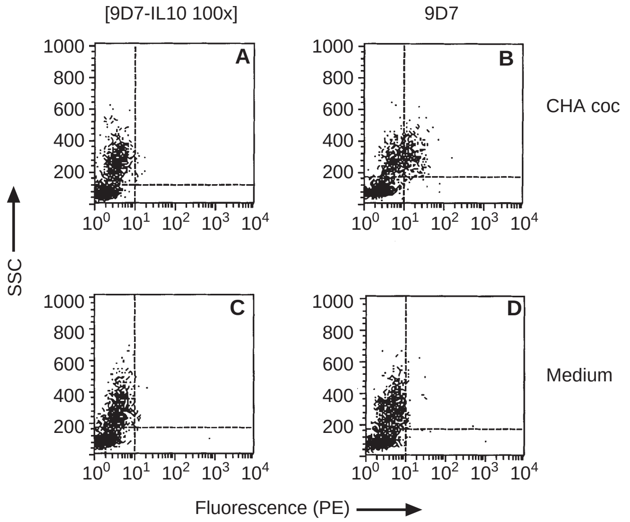

**Figure 4** Intracytoplasmic IL-10 expression by allogeneic PBMCs cultured either in medium or with an RCC cell line (CHA coc as an example) in the presence of monensin (2 μM) was characterized by staining with a PE-conjugated anti-IL-10 antibody (9D7) (**B** and **D**). The specificity of the labelling was assessed by preincubation of 9D7 with a 100-fold excess of IL-10 [9D7IL-10] (**A** and **C**). A control performed with a rat IgG1 control antibody was superimposable on the [9D7-IL-10] labelling (data not shown)

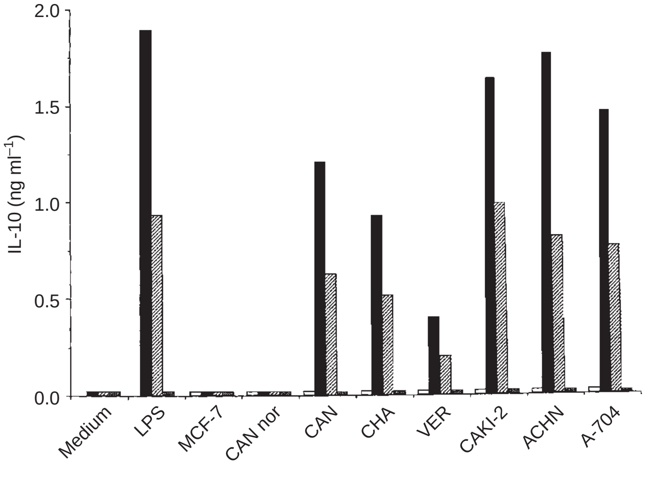

**Figure 3** Mean IL-10 production in co-culture of allogeneic healthy donor PBMCs ($10^6$ cells during 48 h) (■), purified monocytes ($4 \times 10^5$ cells during 48 h) (▨) and lymphocytes ($8 \times 10^5$ cells during 48 h) (▒) with six different RCC cell lines (CAN, CHA, VER, Caki-2, ACHN, A-704), one normal renal epithelial primary culture (CAN nor) and one breast carcinoma cell line (MCF-7). Mean IL-10 production by epithelial cells alone (carcinoma cells or normal renal epithelial cells (□). Positive control was performed using LPS (1 μg ml$^{-1}$). This experiment is representative of five performed with different healthy donor PBMCs. IL-10 secretion by PBMCs (or monocytes) treated with LPS or cultured with RCC is significantly superior to that of PBMCs (or monocytes) co-cultured with breast carcinoma cell lines or normal renal epithelial cell cultures (Student's *t*-test, $P < 0.01$)

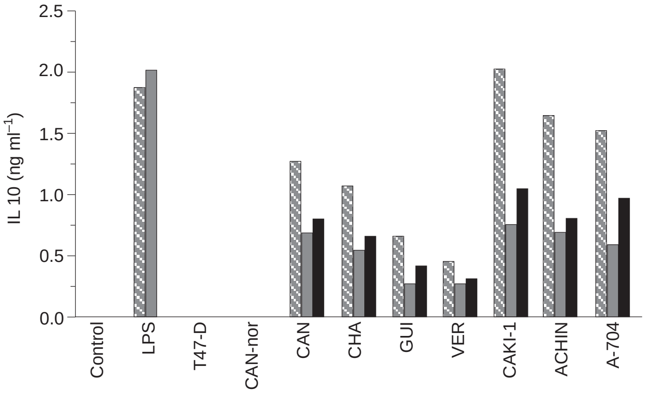

**Figure 5** Mean IL-10 production by allogeneic purified monocytes ($4 \times 10^5$ cells per ml) co-cultured with RCC cell lines ($5 \times 10^4$ cells per well) (▧) or through a permeable membrane of transwell (▩) or with 40% RCC CM (■). This experiment was representative of five performed with different healthy donors

### Flow cytometric analysis

### *Membrane staining*

Flow cytometric analysis was carried out by incubating $5 \times 10^4$–$10^5$ cells for 20 min in 50 μl of phosphate-buffered saline (PBS) with 1% BSA and 0.1% sodium azide on ice with optimal concentrations of the above-mentioned antibodies coupled to phycoerythrin (PE). Cells were washed three times with PBS 1% BSA 0.1% sodium azide. The phenotype of the cells was analysed using a flow cytometer (FACScan).

### *Intracellular staining*

Intracytoplasmic staining of IL-10 was performed after 48 h culture of PBMC with RCC cell lines in presence of Monensin (2 μM) (Sigma) to avoid cytokine secretion. Cells were permeabilized for 5 min in saponin buffer (0.33%) and labelled with an anti-IL-10 rat antibody coupled to PE (9D7) (Pharmingen) for 20 min according to the manufacturer's protocol. After three washes, intracytoplasmic IL-10 expression was analysed on a FACScan.

Labelling specificity was assessed either with a rat IgG control antibody (R & D) staining or by preincubating 9D7 with a 100-fold excess of recombinant IL-10 before PBMC staining.

 *© Cancer Research Campaign 1999*

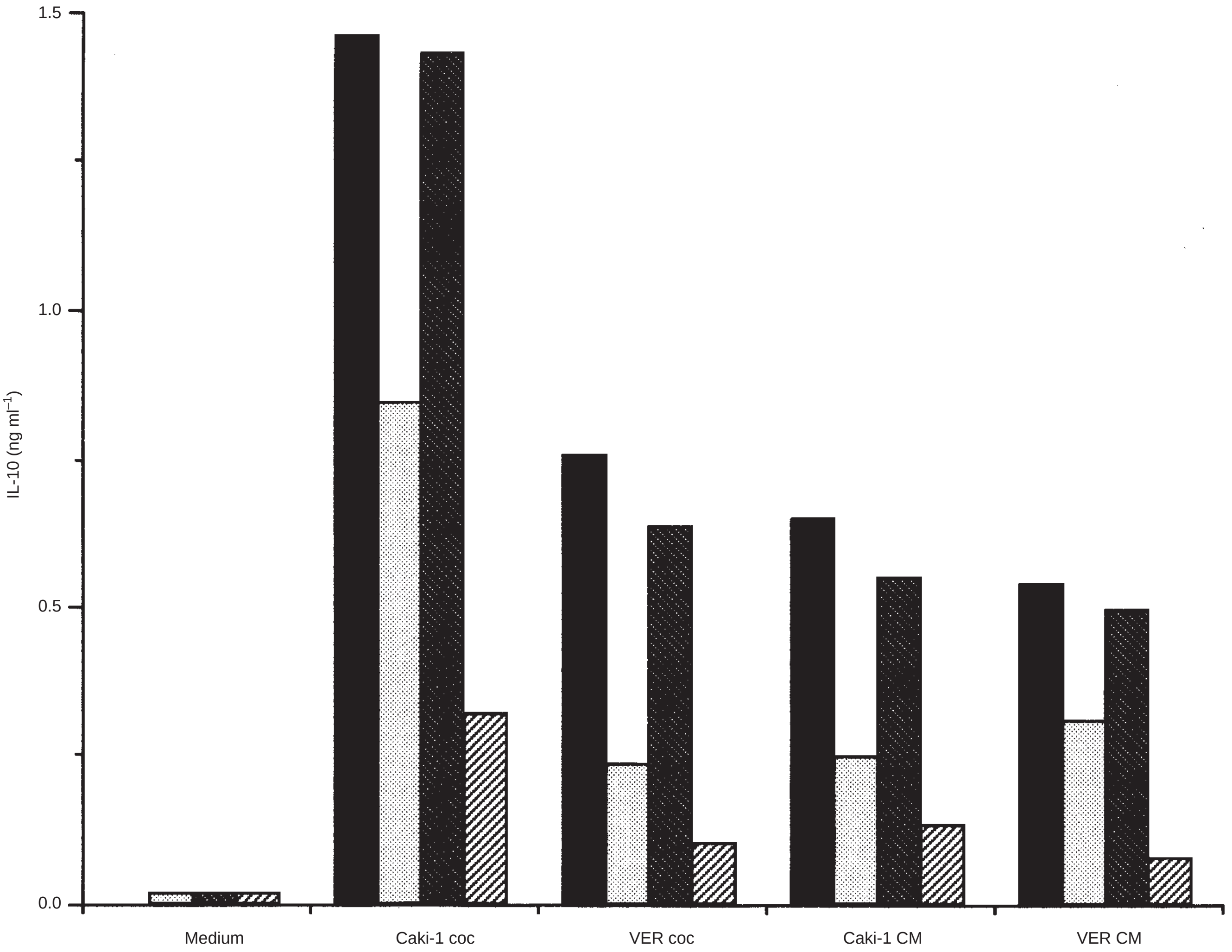

**Figure 6** Mean IL-10 production by purified monocytes cultured with RCC CM (40%) in medium alone (■), with a polyclonal anti-TNF-α Ab (10 μg ml$^{-1}$ (▨), with indomethacin (10 mM) (■) or with the combination of indomethacin +anti-TNF-α Ab (▨). IL-10 production is significantly lower in the presence of anti-TNF-α Ab ($P < 0.01$) but not indomethacin alone ($P > 0.1$). However, IL 10 secretion is significantly lower with anti-TNF-α+indomethacin compared with anti-TNF-α Ab alone in all cell lines (Student's *t*-test, $P < 0.05$)

### Cytokine ELISA

Cell supernatants, collected after 48 h of culture, were tested for the presence of IL-10 using a two-site sandwich ELISA method as previously described (Abrams et al, 1992). The detection limit of IL-10 ELISA is 0.04 ng ml$^{-1}$. TNF-α and $PGE_2$ levels in co-culture supernatants were measured using two-site sandwich immunoassays purchased from Immunotech (Marseille, France) for TNF-α and from Amersham (Les Ulis, France) for $PGE_2$. The sensitivity of the tests was 10 pg ml$^{-1}$, and 16 pg ml$^{-1}$ for TNF-α and $PGE_2$ respectively.

### RT-PCR for IL-10

RT-PCR for IL-10 mRNA was performed as follows: precycle at 94°C for 3 min; cycles 1–40 at 94°C for 1 min (strand preparation), 58°C for 2 min (annealing) and 72°C for 3 min (primer extension). Then, the reaction was held at 72°C for 10 min. All reactions were performed with a Perkin Elmer DNA thermal cycler model 480. The primers for IL-10 mRNA were AGAAGGCATGCACCAGCTCAGCA (3′) and TTTTGGAGACCTCTAATTTATG (5′). Negative control was performed without cDNA adjunction in the master mix reagent. The positive control was the cDNA of the BJAB cell line (kindly provided by N Burdin, Schering Plough Corporation, Dardilly, France).

### Endocytosis

Endocytosis was analysed using a previously described technique (Sallusto et al, 1995). After 48 h of culture, with or without RCC CM or the indicated antibodies and reagent, monocytes were washed three times and resuspended in 10% FCS medium buffered with 25 mM hepes at 37°C. After 10 min, fluorescein isothiocyanate (FITC)–dextran (DX-FITC, Molecular probe, Eugene, OR, USA) was added at the final concentration of 10 μg ml$^{-1}$ for 30 min, at 37°C or at 4°C (control). The cells were then washed four times with cold PBS containing 1% FCS and 0.1% sodium azide and analysed on a FACScan.

© Cancer Research Campaign 1999 

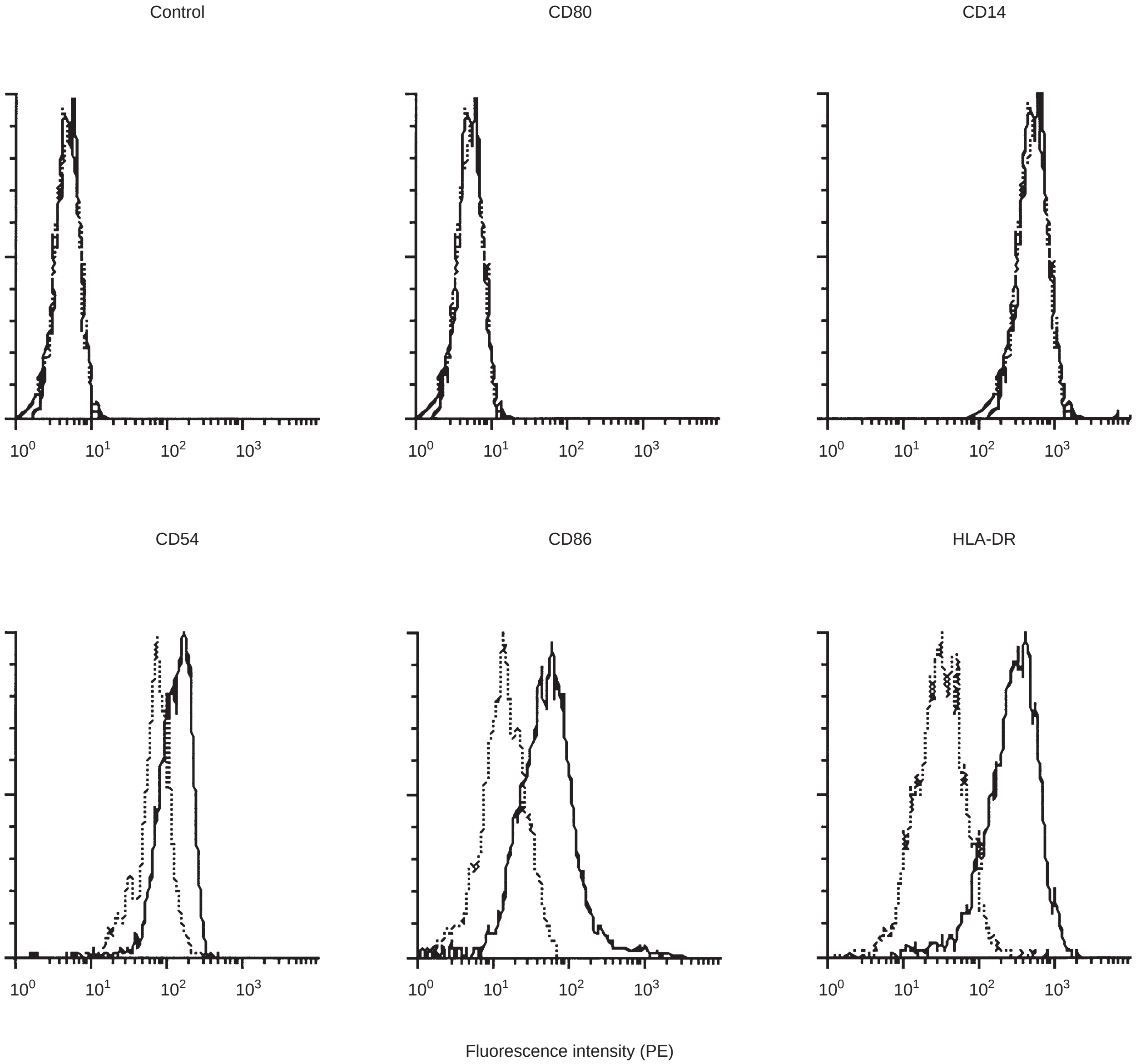

**Figure 7** FACScan analysis of CD14, CD54, CD80, CD86 and HLA-DR expression on monocytes cultured in medium alone (solid line) or with RCC CM (VER) (dotted line). Similar results were obtained with other cell lines (Caki-1, Caki-2, CHA, CAN) and with co-cultures

### Phagocytosis

Monocytes were cultured for 48 h with or without RCC CM or the indicated antibodies and reagent. During the last 4 h of the culture, 0.5 μm latex beads coupled to FITC (1:400) (Polysciences, Warrington, PA, USA) were added to the medium. To analyse the phagocytic capacity of monocytes, cells were recovered and washed three times with PBS. The phagocytosis of the latex beads–FITC was evaluated on a FACScan analyser.

### Statistics

Statistical comparison between samples were performed using Student's *t*-test or Student's paired *t*-test.

### RESULTS

### IL-10 production in renal cell carcinomas

Immunohistochemical analysis of 21 RCC tumour cryostat sections was performed with an anti-IL-10 mAb (Ab 85) using a technique already reported elsewhere (Emilie et al, 1992; Voorzanger et al, 1996). A positive staining for IL-10 was observed in ten of these 21 tumours (47%) (Figure 1A and B). In the surrounding normal renal cell parenchyma, no positive staining for IL-10 was observed (Figure 1G and H). Ten of these 21 RCC tumour samples (including seven IL-10-positive tumours by immunohistochemistry) were found to be positive for IL-10 mRNA expression using RT-PCR, whereas the four non-tumoral

 *© Cancer Research Campaign 1999*

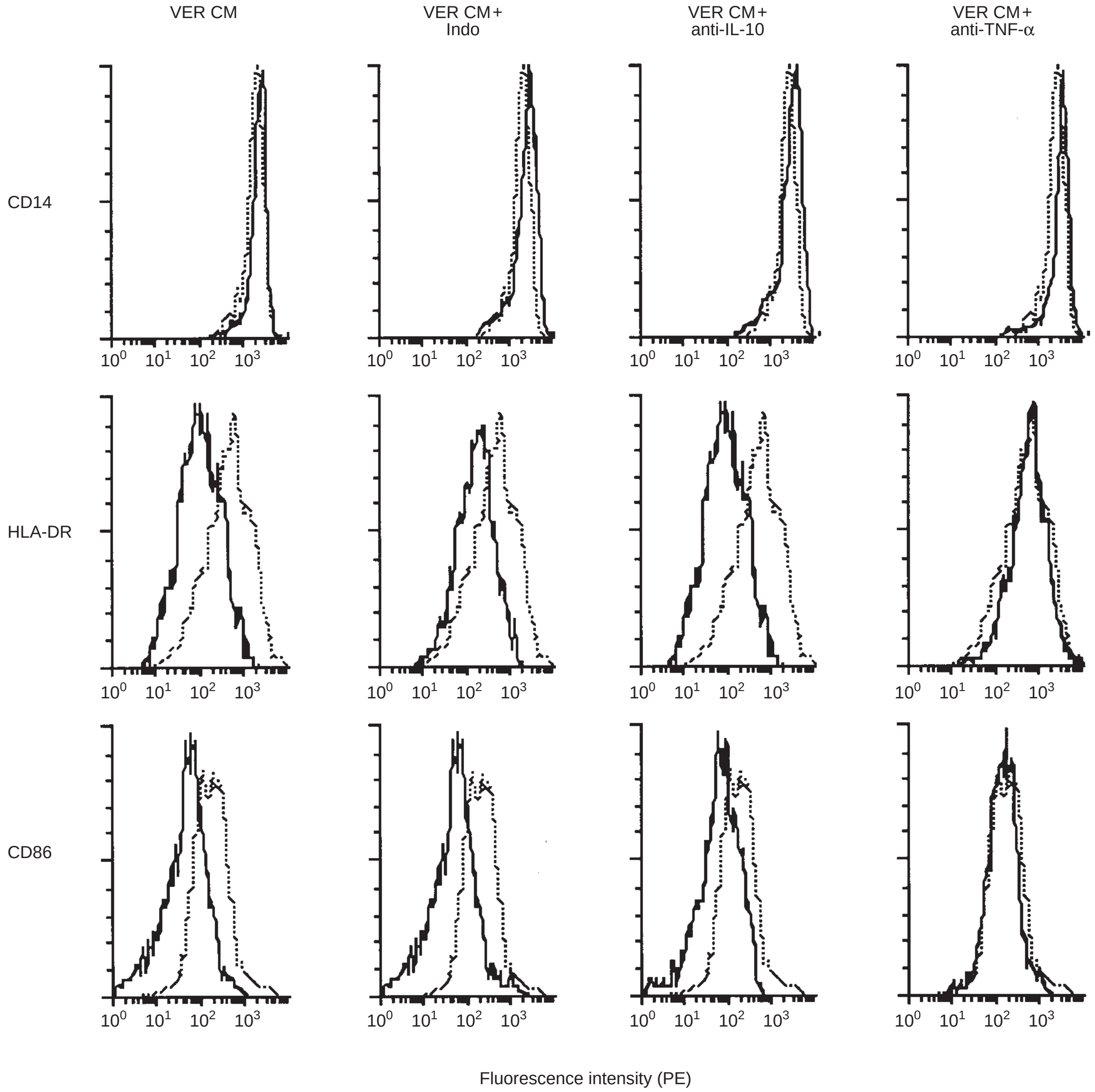

**Figure 8** CD14, HLA-DR and CD86 expression by allogeneic monocytes cultured in medium (dotted line) or with 40% RCC CM (VER as an example) (solid line) alone or in presence of indomethacin (10 μM) or polyclonal antibodies (anti-IL-10 Ab and anti-TNF-α Ab) (10 μg $ml^{-1}$). The mean fluorescence intensity (MFI) of the isotype controls ranged from $10^0$ to $10^1$

kidney samples tested were negative for IL-10 mRNA expression (data not shown). Morphological and topographical examination suggested that IL-10-positive cells are in part infiltrating non-tumoral mononuclear cells (Figure 1B, D and F).

### RCC cell lines induce IL-10 production by monocytes

IL-10 was undetectable in the supernatant of all ten RCC cell lines tested (see Figures 2 and 3 for seven representative cell lines). RCC cell lines were co-cultured for 48 h with autologous PBMCs or allogeneic PBMCs from healthy donors. High levels of IL-10 were detectable in the supernatant of the co-cultures of RCC cell lines with either autologous (Figure 2) or allogeneic PBMCs (Figure 3), as well as in the positive controls, i.e. LPS-treated autologous PBMCs (Figure 2) or allogeneic PBMCs (Figure 3). Of note, RCC-induced IL-10 secretion by autologous PBMCs was lower than that of allogeneic PBMCs in all cell lines tested, possibly because autologous PBMCs were stored frozen prior to the experiment, whereas allogeneic PBMCs were collected and used immediately (Figures 2 and 3). In contrast, IL-10 was undetectable in the supernatant of the primary culture of normal renal epithelial cells (CAN nor) co-incubated with autologous (Figure 2) or allogeneic PBMCs (Figure 3). Similarly, IL-10 was undetectable in co-cultures of allogeneic PBMCs with breast cell carcinoma cell lines (Figure 3). Purified lymphocytes (< 3% $CD14^+$) failed to produce IL-10 in the presence of LPS or RCC cell lines (Figure 3), whereas purified monocytes (85–90% $CD14^+$) produced IL-10 in both of these conditions (Figure 3). Intracytoplasmic staining with an anti-IL-10 antibody (9D7) demonstrated that only monocytes secreted IL-10 under stimulation with RCC cell lines (Figure 4B). The specificity of this staining was assessed by preincubation of anti IL-10 with a 100-fold excess of recombinant IL-10 before PBMC staining (Figure 4A). Monocytes also produced IL-10 when cultured with RCC cell

© Cancer Research Campaign 1999

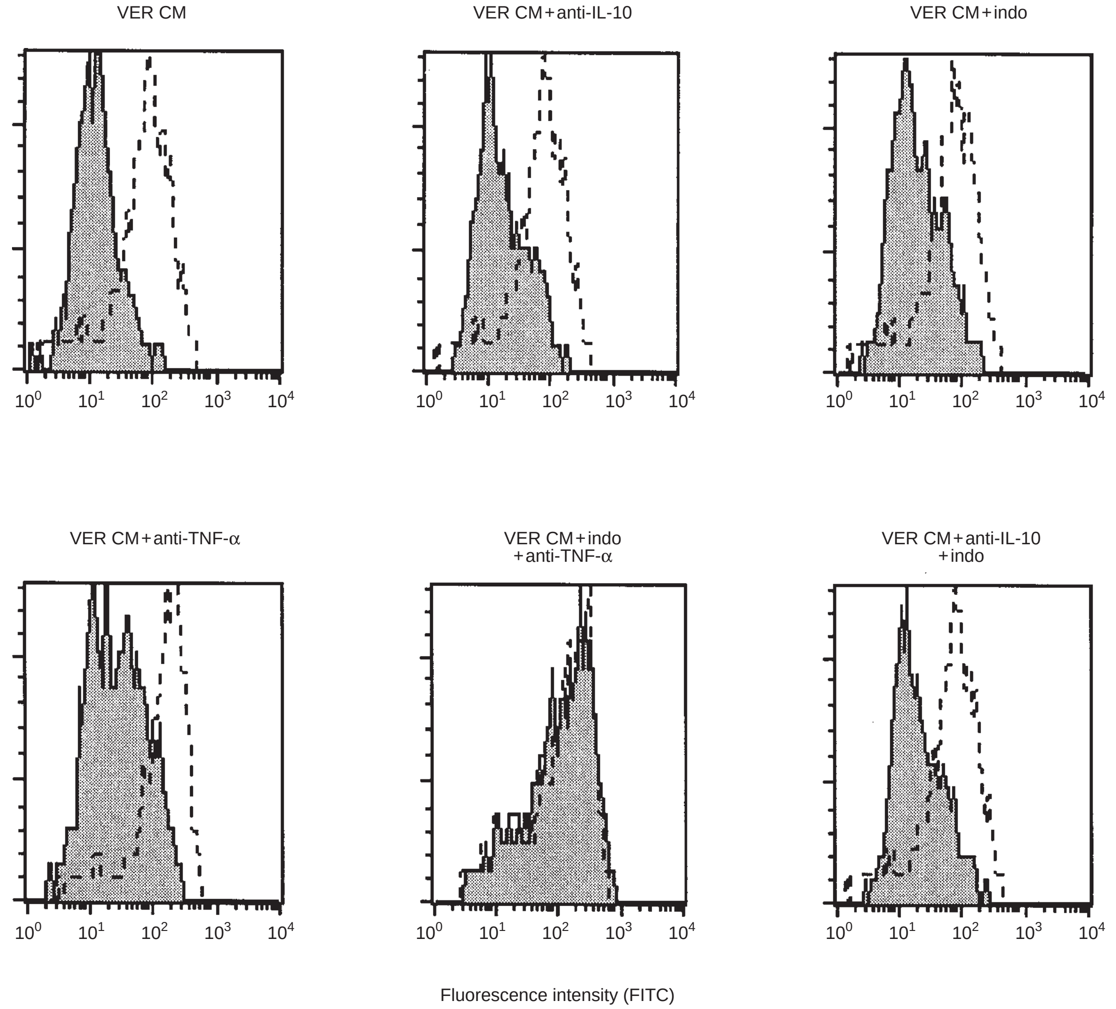

**Figure 9** Endocytic capacity (mannose receptor mediated) of monocytes evaluated by dextran–FITC (DX–FITC) incorporation (10 µg ml$^{-1}$)0 at 37°C for 30 min. Comparison of endocytosis of DX–FITC in medium (dotted line) or in presence of 40% RCC CM (VER as an example) (solid line) alone or in the presence of polyclonal antibodies (anti-TNF-α Ab and anti-IL-10 Ab) (10 µg ml$^{-1}$) or indomethacin (10 µM). A non-related polyclonal antibody (see Materials and methods) had no effect on endocytic capacity (not shown)

lines in a transwell system or in the presence of RCC CM, indicating that IL-10 production was induced at least in part by a soluble factor produced by RCC cell lines (Figure 5). IL-10 production correlated with the percentage of RCC CM added in the culture, e.g. < 0.04 ng ml$^{-1}$ with 5% RCC CM, 0.328 ng ml$^{-1}$ with 10%, 0.619 ng ml$^{-1}$ with 20%, 0.88 ng ml$^{-1}$ with 40% and 1.02 ng ml$^{-1}$ with 80% in a representative experiment with the conditioned medium of the CAN cell line. All subsequent experiments were performed with allogeneic monocytes cultured with 40% RCC CM.

### IL-10 production is mediated by TNF-α and $PGE_2$

TNF-α and $PGE_2$ have been reported to induce IL-10 production by monocytes and macrophages (Wanidworanum and Strober, 1993; Kambayashi et al, 1995). Heat inactivation (100°C, 30 min) of RCC CM reduced partially (32% to 60%) IL-10 production by the eight different RCC CM tested, suggesting the co-involvement of a heat-insensitive molecule. Indeed, high levels of TNF-α and $PGE_2$ were detectable in the supernatant of monocytes cultured in the presence of RCC but not with breast carcinoma conditioned medium (Table 1). A polyclonal anti-TNF-α Ab blocked 40–70% of the production of IL-10 by monocytes incubated with the RCC CM of CAKI-1 and VER whereas indomethacin (an inhibitor of PGE-2 synthesis) induced a minor inhibition of IL-10 production (Figure 6). Anti-TNFα Ab significantly reduced the production of PGE-2 by monocytes incubated with Caki-1 or VER CM (70.8 ± 12%; range = 57%–85%, $P < 0.05$ using Student's *t*-test in three experiments), indicating that $PGE_2$ production by monocytes is in part induced by TNF-α. The combination of anti-TNF-α Ab and indomethacin inhibited, at least additively, IL-10 production (80–94%) (Figure 6). These results indicate that TNF-α and $PGE_2$ are responsible for the induction of IL-10 production by monocytes incubated in the presence of RCC CM.

Importantly, the supernatant of the six RCC lines tested contained only low or undetectable TNF-α levels and no $PGE_2$ (Table 1). This suggests that RCC cell lines produce a soluble mediator which induces TNF-α and $PGE_2$ production by monocytes, both molecules acting additively to induce IL-10 production through an autocrine loop.

 *© Cancer Research Campaign 1999*

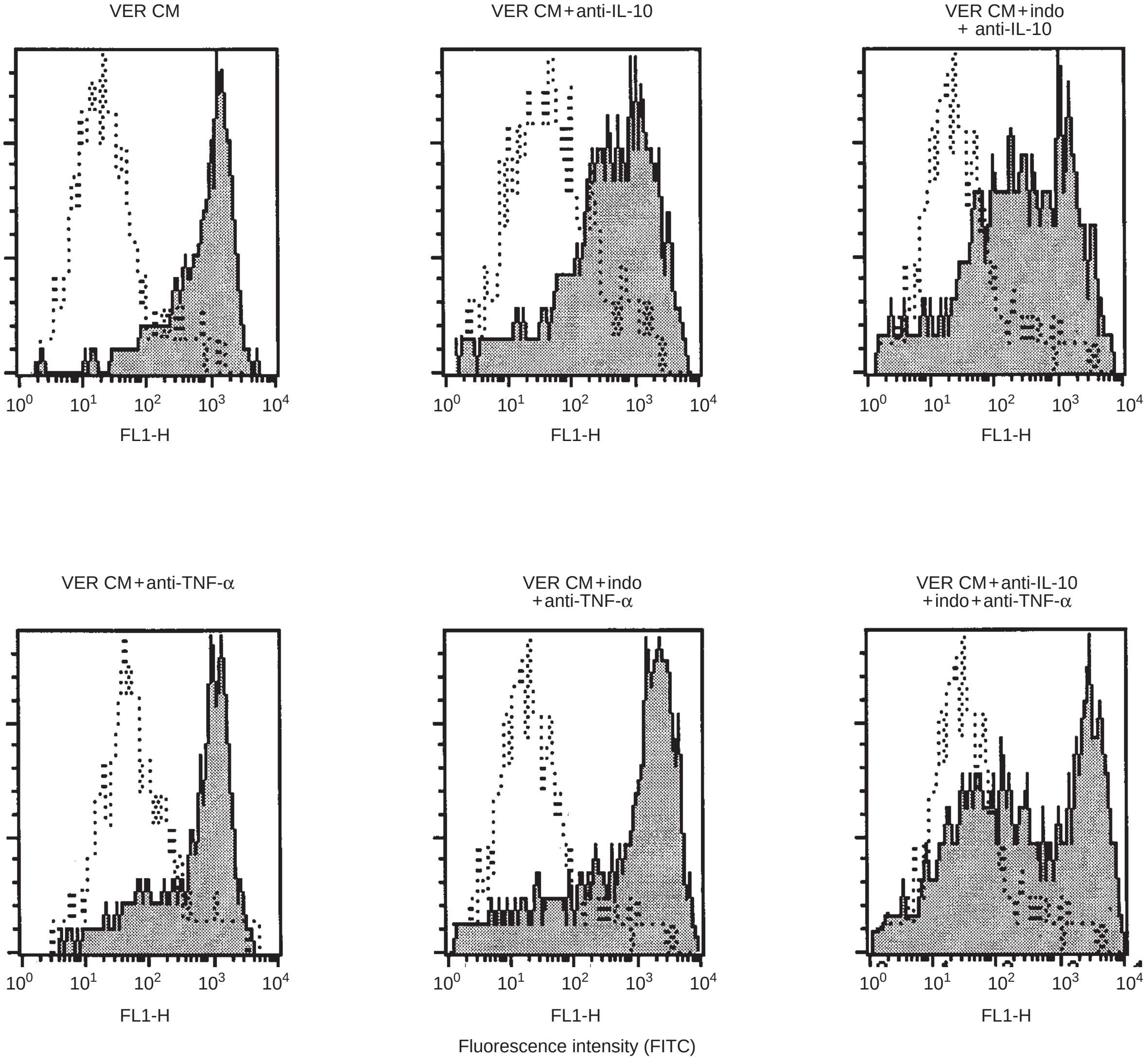

**Figure 10** Phagocytosis of latex beads (0.5 μm) coupled to FITC by monocytes cultured in medium (dotted line) or in the presence of 40% RCC CM (VER as an example) (solid line) alone or in the presence of indomethacin (10 μM) or polyclonal antibodies (anti-TNF-α Ab and anti-IL-10 Ab) (10 μg ml$^{-1}$). A non-related polyclonal antibody (see Materials and methods) had no effect on phagocytic capacity (not shown)

### Phenotypic and functional alterations of monocytes by RCC: role of TNF-$\alpha$, $PGE_2$ and IL-10

### *Phenotypic modifications*

Monocytes were collected after 2 days of culture with RCC CM and tested for CD14, CD54, CD80, CD86 and HLA-DR expression. The conditioned medium of the five RCC cell lines tested (Caki-1, Caki-2, CHA, VER, CAN), but not of breast carcinoma cell lines (T47-D, MCF-7, SK-BR3) (not shown), induced a significant decrease in HLA-DR, CD54 and CD86 expression, whereas CD14 an CD80 expression were unaffected (Figure 7). Anti-IL-10 Ab, but not a control polyclonal Ab, partially reversed the inhibition of CD86 expression induced by RCC CM, without affecting HLA-DR expression. Indomethacin (10 μM) partially reversed the decrease in HLA-DR expression without affecting CD86 expression (Figure 8). The decrease in CD86 and HLA-DR expression induced by RCC CM could be completely abrogated by the addition of an anti-TNF-α neutralizing Ab (Figure 8) whereas the polyclonal control Ab had no effect (not shown).

### *Modulation of endocytosis and phagocytosis capacities by RCC CM*

The endocytic capacity of monocytes cultured in RPMI–10% FCS for 48 h was evaluated using dextran–FITC incorporation (Figure 9), which was inhibited by incubation at 4°C or with a solution of mannane (0.3 mg ml$^{-1}$) (data not shown). The addition of RCC CM (VER and Caki-1 cell lines), but not breast carcinoma CM (data not shown), completely inhibited the endocytic capacity of monocytes (Figure 9). This effect was partially reversed by an anti-IL-10 Ab, and completely reversed by an anti-TNF-α Ab, whereas polyclonal control Ab had no effect (Figure 9).

In contrast, the capacity of monocytes to phagocyte 0.5 μm latex beads coupled to FITC was strongly enhanced in presence of the RCC CM (VER and Caki-1 cell lines) (Figure 10). Anti-IL-10 Ab alone partially abrogated this effect of RCC CM (Figure 10). Although indomethacin and anti-TNF-α alone had no effect, the combination of anti-IL-10 Ab with indomethacin + anti-TNF-α Ab synergistically blocked the increase in phagocytic capacity mediated by RCC CM.

© Cancer Research Campaign 1999

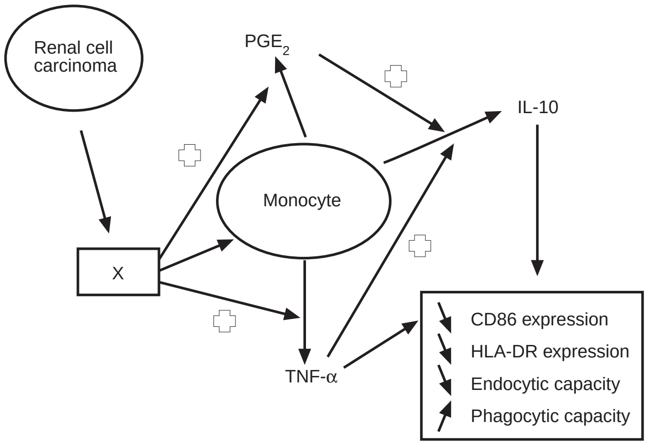

**Figure 11** A proposed scheme for the cytokine cascade elicited by RCC

### DISCUSSION

The objectives of this study were to investigate the production by RCC tumours of immunosuppressive factors that may potentially affect the anti-tumour immune response. The results presented show that IL-10, an immunosuppressive cytokine, is detectable by immunohistochemistry in RCC tumours but not produced by RCC cells purified from these biopsies. The production of IL-10 by non-tumoral cells in biopsies has also been reported in other tumour models such as non-Hodgkin's lymphoma (Voorzanger et al, 1996). However, RCC cell lines, but not normal renal epithelial cells or breast carcinoma cell lines, were found to produce (a) soluble factor(s) which triggers the production of IL-10 by autologous and allogeneic monocytes in vitro.

These results are in agreement with previous observations showing that IL-10 transcripts are detectable in tumour-infiltrating leucocytes of RCC tumours by RT-PCR (Filgueira et al, 1993; Mauerer et al, 1995; Nakagomi et al, 1995; Wang et al, 1995). However, in this model, monocytes were found to be the major IL-10 producers among PBMCs, whereas purified lymphocytes failed to produce IL-10 in the same conditions. The observation that normal renal epithelial cells are not able to induce IL-10 production in vitro is consistent with the absence of IL-10 production by the normal adjacent renal parenchyma and indicates that the capacity to induce IL-10 production is highly correlated with a malignant phenotype for renal epithelial cells.

It is also important to notice that IL-10 production was significantly higher when monocytes and tumoral cells were in the same well as compared with culture in a transwell system, without contact between monocytes and tumoral cells. This observation could be explained by greater concentration of soluble factors at the contact of RCC cells, allowing the strongest activation of monocytes. However, we cannot exclude the possibility that a transmembrane molecule expressed at the surface of RCC cells may contribute to stimulate monocyte production of TNF-α and IL-10.

The results presented here show that RCC CM also elicited the production of $PGE_2$ and TNF-α by monocytes and that anti-TNF-α Ab and indomethacin, an inhibitor of $PGE_2$ production, at least additively inhibited IL-10 production induced by RCC CM. TNF-α and $PGE_2$ are well-known inducers of IL-10 production (Wanidworanum and Strober, 1993; Kambayashi et al, 1995; Huang et al, 1996) and recombinant TNF-α and $PGE_2$ were found to act synergistically in the induction of IL-10 production by monocytes in vitro (not shown). However, the capacity of indomethacin to affect IL-10 production has not been reported. Although a role for other metabolites of arachidonic acid cannot be excluded, these results strongly suggest that TNF-α and $PGE_2$ are involved in the induction of IL-10 production by monocytes elicited by RCC CM. An anti-TNF-α Ab was found capable of blocking RCC-mediated $PGE_2$ production by monocytes, in agreement with previous observations showing that TNF-α induces $PGE_2$ production (Bachwich et al, 1986; Lehmmann et al, 1988; Alleva et al, 1993). Taken together, these results indicate that TNF-α is a central mediator for the induction of $PGE_2$ and IL-10 production by monocytes in this model. The TNF-α/$PGE_2$/IL-10 cascade is induced by RCC cells but not by normal renal epithelial cells or by the breast carcinoma cell lines tested.

In the present study, the addition of RCC CM potently affected the phenotype and function of peripheral blood monocytes cultured in vitro. RCC CM induced a down-regulation of both CD86 and HLA-DR expression on monocytes, these phenomena being reversed partially by an anti-IL-10 Ab and indomethacin respectively. This suggests that RCC CM-mediated inhibition of HLA-DR and CD86 expression is in part mediated by $PGE_2$ and IL-10 respectively. Indeed, $PGE_2$ and recombinant IL-10 respectively block HLA-DR and CD86 expression in vitro (data not shown), in agreement with previous reports (de Waal Malefyt et al, 1991). However, indomethacin may also block other arachidonic acid mediators that might play a role in this phenomenon. Although the anti-IL-10 Ab tested was capable of antagonizing the inhibition of CD86 induced by similar concentrations of recombinant IL-10 (100 U $ml^{-1}$) (not shown), it failed to abrogate the effect of RCC CM, suggesting the existence of other inhibitory factors elicited by RCC CM. Indeed, an anti-TNF-α Ab abrogated the down-regulation of CD86 and HLA-DR expression induced by RCC CM much more efficiently than anti-IL-10 Ab or indomethacin. To our knowledge, this effect of an anti-TNF-α Ab has not been previously described. Anti-TNF-α may act here by inhibiting IL-10 and $PGE_2$ production by monocytes, which may result in a more efficient inhibition than the $PGE_2$ and IL-10 inhibitors used in this study.

In addition to these phenotypic alterations, the supernatant of RCC cell lines induced a strong down-regulation of endocytosis mediated by mannose receptor, a phenomenon that was also partly abrogated by an anti-IL-10 and completely inhibited by anti-TNF-α Ab. Endocytosis is an initial step allowing exogenous antigen processing within the endocytic compartment, followed by antigen presentation by MHC class II receptors (for review see Watts, 1997). The loss of CD86 and HLA-DR expression on monocytes induced by RCC CM may have important functional consequences for the immune response against RCC cells. In vivo, in man, the lack of expression of HLA class II antigens in RCC tumours has been associated with a lack of response to immunotherapy with IL-2 (Cohen et al, 1987; Rubin et al, 1989). The results presented here suggested that the loss of HLA-DR expression may be induced by RCC tumour cells themselves. The simultaneous inhibition of mannose receptor-mediated endocytosis by the TNF-α/$PGE_2$/IL-10 cascade induced by RCC CM may further contribute to impair the antigen presentation properties of monocytes in vivo.

Finally, in contrast to these inhibitory properties, RCC CM were found to increase the phagocytic capacity of monocytes, an effect

 © Cancer Research Campaign 1999

which was partially blocked by anti-IL-10 Ab. Although indomethacin (not shown) and anti-TNF-α had no effect, the combination of anti-IL-10 Ab, indomethacin and anti-TNF-α Ab synergistically blocked the increase in phagocytic activity. This synergistic effect may result from the inhibition of IL-10 production induced by anti-TNF-α and indomethacin. This is in agreement with recent reports showing that IL-10 increases the phagocytic capacity of macrophages (Capsoni et al, 1995; Hashimoto et al, 1997).

The soluble factor(s) responsible for the activation of the TNF-α/$PGE_2$/IL-10 cascade are not yet characterized. RCC CM contain only low or undetectable levels of these mediators, indicating that RCC cell lines produce (a) soluble mediator(s) capable of inducing the production of TNF-α by monocytes (Figure 11). The factor(s) responsible for the cytokine cascade induced by RCC CM is (are) currently under investigation since cytokines previously reported to be produced by RCC, i.e. TGF-$\beta_1$ FGFb, IL-6, IL-8, TGF-α and GM-CSF, were found unable to induce IL-10 production by monocytes (not shown).

Taken together, these results indicate that RCCs induce the production of a cytokine cascade TNF-α/$PGE_2$/IL-10 which inhibits the expression of surface molecules involved in the antigen-presenting function of monocytes as well as their mannose receptor-mediated endocytic capacity. These phenomena may interfere with the spontaneous and therapeutic immune response against RCC tumours in vivo in patients.

### ACKNOWLEDGEMENTS

This work was supported by grants from Le comité de Saône et Loire de la Ligue contre le Cancer, la Ligue Nationale contre le Cancer and the Association pour la Recherche sur le Cancer (ARC).

### REFERENCES

Abrams JS, Roncarolo MG, Yssel H, Andersson U, Gleich GJ and Silver JE (1992) Strategies of anti-cytokine monoclonal antibody development: immunoassay of IL-10 and IL-5 in clinical samples. *Immunol Rev* **127**: 5–24

Alleva DG, Askew D, Burger CJ and Elgert KD (1993) Tumor-induced macrophage Tumor Necrosis Factor-α production suppresses autoreactive T cells. *Immunobiology* **188**: 430–435

Bachwich PR, Chensue SW, Larrick JW and Kunkel SL (1986) Tumor necrosis factor α stimulates interleukin-1 and prostaglandin-E2 production in resting macrophages. *Biochem Biophys Res Commun* **136**: 94–101

Bain C, Puisieux I, Merrouche Y, Duc A, Colombo MP and Favrot MC (1996) B7.1 gene transduction of human renal-cell-carcinoma cell lines restores the proliferative response and cytotoxic function of allogeneic T cells. *Int J Cancer* **67**: 769–776

Benjamin D, Knoblock TJ and Dayton MA (1992) Human B cell interleukin-10: B cell lines derived from patients with acquired immunodeficiency syndrome and Burkitt's lymphoma constitutively secrete large quantities of interleukin-10. *Blood* **80**: 1289–1298

Blay JY, Burdin N, Rousset F, Lenoir G, Biron P, Philip T, Banchereau J and Favrot MC (1993) Serum interleukin-10 in non-Hodgkin's lymphoma: a prognostic factor. *Blood* **82**: 2169–2174

Capsoni F, Minonzio F, Ongari AM, Carbonelli V, Galli A and Zanussi C (1995) IL-10 up-regulates human monocyte phagocytosis in the presence of IL-4 and IFNγ. *J Leukoc Biol* **58**: 351–358

Cohen PJ, Lotze MT, Roberts JR, Rozenberg S and Jaffé ES (1987) The immunopathology of sequential tumor biopsies in patients treated with IL2: correlation of response with T cell infiltration and HLA-DR expression. *Am J Pathol* **129**: 208–216

Combaret V, Wang Q, Favrot MC, Thiesse P, Philip I, Bouffet E, Bailly C, Bouvier R, Chauvin F, Zucker JM, Bernard JL, Lenoir G and Philip T (1989) Clinical value of N-myc oncogene amplification in 52 patients with neuroblastoma included in recent therapeutic protocols. *Eur J Clin Oncol* **24**: 1607–1612

de Waal Malefyt R, Abrams J, Bennett B, Figdor CG and de Vries JE (1991) Interleukin 10 (IL-10) inhibits cytokine synthesis by human monocytes: an autoregulatory role of IL-10 produced by monocytes. *J Exp Med* **174**: 1209–1220

de Waal Malefyt R, Yssel H and de Vries JE (1993) Direct effect of IL-10 on subsets of human $CD4^+$ T cell clones and resting T cells. Specific inhibition of IL-2 production and proliferation. *J Immunol* **150**: 4754–4765

Emilie D, Touitou R, Raphael N, Peuchmaur N, Devergnee O, Coumharas DRJ, Crevon NC, Edelman L, Jouab I and Galanaud P (1992) In vivo production of interleukin-10 by malignant cells in AIDS lymphoma. *Eur J Immunol* **22**: 2937–2942

Filgueira L, Zuber M, Merlo A, Caetano V, Schultz E, Harder F, Spagnoli GC and Heberer M (1993) Cytokine gene transcription in renal cell carcinoma. *Br J Surg* **80**: 1322–1325

Fiorentino DF, Zlotnik A, Vieira P, Mosmann TR, Howard M, Moore KW and O'Garra A (1991) IL-10 acts on the antigen-presenting cell to inhibit cytokine production by Th1 cells. *J Immunol* **146**: 3444–3451

Gastl GA, Abrams JS, Nanus DM, Oosterkamp R, Silver J, Liu F, Chen M, Albino AP and Bander NH (1993) Interleukin-10 production by human carcinoma cell lines and its relationship to interleukin-6 expression. *Int J Cancer* **55**: 96–101

Groux H, O'Garra A, Bigler M, Rouleau M, Antonenko S, De Vries JE and Roncarolo MG (1997) A $CD4^+$ T-cell subset inhibits antigen-specific T-cell responses and prevents colitis. *Nature* **389**: 737–742

Hashimoto S, Yanada M, Motoyoshi K and Akagawa KS (1997) Enhancement of macrophage colony stimulating factor induced growth and differentiation of human monocytes by IL-10. *Blood* **89**: 315–321

Howard M and O'Garra A (1992) Biological properties of interleukin 10. *Immunol Today* **12**: 198–201

Huang M, Sharma S, Mao JT and Dubinet SM (1996) Non small lung cancer-derived soluble mediators and prostaglandin $E_2$ enhance peripheral blood lymphocyte IL-10 transcription and protein production. *J Immunol* **157**: 5512–5520

Kambayashi T, Alexander R, Fong M and Strassmann G (1995) Potential involvement of IL-10 in suppressing tumor-associated macrophages. Colon 26 derived PGE-2 inhibits TNFα release via a mechanism involving IL-10. *J Immunol* **154**: 3383–3390

Lehmmann V, Benninghoff B and Droge W (1988) Tumor necrosis factor α induced activation of peritoneal macrophages is regulated by Prostaglandin-E2 and cAMP. *J Immunol* **141**: 587–591

Mauerer MJ, Martin DM, Castelli C, Elder E, Leder G, Storkus WJ and Lotze MT (1995) Host immune response in renal cell cancer: interleukin-4 (IL-4) and IL-10 mRNA are frequently detected in freshly collected tumor-infiltrating lymphocytes. *Cancer Immunol Immunother* **41**: 111–121

Nakagomi H, Pisa P, Pisa EK, Yamamoto Y, Halapi E, Backlin K, Juhlin C and Kiessling R (1995) Lack of interleukin-2 (IL-2) expression and selective expression of IL-10 mRNA in human renal cell carcinoma. *Int J Cancer* **63**: 366–371

Négrier S, Escudier B, Lasset C, Savary J, Chevreau C, Ravaud A, Peny J and Mousseau M (1996) The FNCLCC CRECY trial: interleukin 2 (IL2) + interferon (IFN)-α is the optimal treatment to induce responses in metastatic renal cell carcinoma (MRCC). *Proc Am Soc Clin Oncol* **15**: 248 (abstract)

Oosterwijk E, Ruiter DJ, Hoedemaeker PJ, Pauwels EKJ, Jonas U, Zwartendijk J and Warnaar SO (1986) Monoclonal antibody G-250 recognizes a determinant present in renal cell carcinoma and absent from normal kidney. *Int J Cancer* **38**: 489–494

Pisa P, Halapi E, Pisa EK, Gerdin E, Hising C, Bucht A and Kiessling R (1992) Selective expression of interleukin-10, interferon γ, and granulocyte–macrophage colony stimulating factor in ovarian cancer biopsies. *Proc Natl Acad Sci* (USA) **89**: 7708–7712

Rosenberg SA, White DE, Lotze MT, Yang JC, Linehan WM, Seipp C, Calabro S, Karp SE, Sherry RM, Steinberg S and White DE (1989) Combination therapy with interleukin-2 and α interferon for the treatment of patients with advanced cancer. *J Clin Oncol* **7**: 1863–1874

Rubin JT, Elwood LJ, Rosenberg SA and Lotze MT (1989) Immunohistochemical correlates of response to recombinant Interleukin-2-based immunotherapy in humans. *Cancer Res* **49**: 7086–7092

Sallusto F and Lanzavecchia A (1994) Efficient presentation of soluble antigen by cultured human dendritic cells is maintained by granulocyte/macrophage colony stimulating factor plus interleukin 4 and down regulated by tumor necrosis factor α. *J Exp Med* **179**: 1109–1118

Sallusto F, Cella M, Danieli C and Lanzavecchia A (1995) Dendritic cells use macropinocytosis and the mannose receptor to concentrate macromolecules in the major histocompatibility complex class II compartment: down regulation by cytokines and bacterial products. *J Exp Med* **182**: 389–400

© Cancer Research Campaign 1999

Taga K, Mostowski H and Tosato G (1993) Human interleukin 10 can directly inhibit T cell growth. *Blood* **81**: 2964–2971
Voorzanger N, Touitou R, Delecluse HJ, Rousset F, Joab I, Favrot MC and Blay JY (1996) IL-10 and IL-6 are produced in vivo by non Hodgkin's lymphoma cells and act as cooperative growth factors. *Cancer Res* **56**: 5499–5505
Wang Q, Redovan C, Tubbs R, Olencki T, Klein E, Kudoh S, Finke J and Bukowski RM (1995) Selective cytokine gene expression in renal cell carcinoma tumor cells and tumor infiltrating lymphocytes. *Int J Cancer* **61**: 780–785
Wanidworanum C and Strober W (1993) Predominant role of tumor necrosis factor-α in human monocyte IL-10 synthesis. *J Immunol* **151**: 6853–6861
Watts C (1997) Capture and processing of exogenous antigens for presentation on MHC molecules. *Annu Rev Immunol* **15**: 821–850

 *© Cancer Research Campaign 1999*
